# Non-invasive assessment of retinal alterations in mouse models of infantile and juvenile neuronal ceroid lipofuscinosis by spectral domain optical coherence tomography

**DOI:** 10.1186/2051-5960-2-54

**Published:** 2014-05-10

**Authors:** Janos Groh, David Stadler, Mathias Buttmann, Rudolf Martini

**Affiliations:** Department of Neurology, Developmental Neurobiology, University of Wuerzburg, Josef-Schneider-Str. 11, D-97080 Wuerzburg, Germany; Department of Neurology, University of Wuerzburg, Josef-Schneider-Str. 11, D-97080 Wuerzburg, Germany

**Keywords:** Optical coherence tomography, Neuronal ceroid lipofuscinosis, Neurodegeneration, Retinal degeneration, Lysosomal storage disease

## Abstract

**Introduction:**

The neuronal ceroid lipofuscinoses constitute a group of fatal inherited lysosomal storage diseases that manifest in profound neurodegeneration in the CNS. Visual impairment usually is an early symptom and selective degeneration of retinal neurons has been described in patients suffering from distinct disease subtypes. We have previously demonstrated that palmitoyl protein thioesterase 1 deficient (*Ppt1*^*-/-*^) mice, a model of the infantile disease subtype, exhibit progressive axonal degeneration in the optic nerve and loss of retinal ganglion cells, faithfully reflecting disease severity in the CNS. Here we performed spectral domain optical coherence tomography (OCT) in *Ppt1*^*-/-*^ and ceroid lipofuscinosis neuronal 3 deficient (*Cln3*^*-/-*^) mice, which are models of infantile and juvenile neuronal ceroid lipofuscinosis, respectively, in order to establish a non-invasive method to assess retinal alterations and monitor disease severity *in vivo*.

**Results:**

Blue laser autofluorescence imaging revealed increased accumulation of autofluorescent storage material in the inner retinae of 7-month-old *Ppt1*^*-/-*^ and of 16-month-old *Cln3*^*-/-*^ mice in comparison with age-matched control littermates. Additionally, optical coherence tomography demonstrated reduced thickness of retinae in knockout mice in comparison with age-matched control littermates. High resolution scans and manual measurements allowed for separation of different retinal composite layers and revealed a thinning of layers in the inner retinae of both mouse models at distinct ages. OCT measurements correlated well with subsequent histological analysis of the same retinae.

**Conclusions:**

These results demonstrate the feasibility of OCT to assess neurodegenerative disease severity in mouse models of neuronal ceroid lipofuscinosis and might have important implications for diagnostic evaluation of disease progression and therapeutic efficacy in patients. Moreover, the non-invasive method allows for longitudinal studies in experimental models, reducing the number of animals used for research.

## Introduction

The assessment of neuronal perturbation and neurodegeneration is of critical importance in many diseases of the central nervous system (CNS), as it strongly correlates with permanent neurological disability. Therefore, minimally- or non-invasive techniques for unbiased determination of neuronal perturbation are not only useful for diagnosis, but even more so for monitoring disease progression and efficacy of therapeutic interventions.

An ideal CNS-related target to monitor these issues is the eye with its easy accessibility. Consequently, optical coherence tomography (OCT) has emerged as an important diagnostic tool not only to monitor ophthalmological disorders but also other neurological diseases of the CNS including multiple sclerosis, Alzheimer’s and Parkinson’s disease [1–3]. The neuronal ceroid lipuscinoses (CLN diseases; NCLs), a group of fatal neurodegenerative lysosomal storage diseases, may comprise additional morbidities that can be diagnosed and followed up by OCT, as the visual system is often affected early during pathogenesis and might reflect disease stage and progression in other regions of the CNS.

The different CLN subtypes are caused by mutations in distinct *CLN* genes encoding proteins important for lysosomal function and cellular homeostasis [4]. Deficiencies in these proteins result in the accumulation of lipofuscin-like autofluorescent storage material (also termed ceroid) in the cytoplasm of neurons and other cell types and a selective loss of neurons in the brain and retina, eventually culminating in disability and early death [5, 6].

Deficiencies in the soluble lysosomal enzyme palmitoyl protein thioesterase 1 (PPT1) result in the devastating infantile form of the disease (CLN1 disease) and mutations in ceroid lipofuscinosis neuronal 3 (CLN3), a transmembrane protein of uncertain function, are cause of the most common juvenile subtype (CLN3 disease) [7]. In both subtypes, visual impairment is often one of the first symptoms, and retinal degeneration has been described in patients, but the cause and progression of visual deficits is not well understood [8–11].

Mouse models of CLN1 and CLN3 disease have been generated using different genetic strategies and authentically mimic human disease aspects [12–16]. Retinal dysfunction and retinal degeneration have previously been investigated in these mouse models. Taken together, histological and electrophysiological studies in *Ppt1* and *Cln3* mutant mice have demonstrated that deficits mainly occur in the optic nerve and inner retinal layers at distinct ages [17–23]. Accordingly, impairment of visual acuity also affects mouse models of CLN1 and CLN3 at distinct ages [18, 24].

In order to monitor retinal degeneration in a non-invasive manner, we performed spectral domain optical coherence tomography (OCT) in *Ppt1*^*-/-*^ and *Cln3*^*-/-*^ mice. By combining confocal laser fundus imaging and cross-sectional OCT analysis with high resolution, we demonstrate an accumulation of autofluorescent storage material and a thinning of distinct retinal layers indicative of neurodegeneration. We show that neuron loss in both mouse models predominantly affects the inner retina and confirm that these features occur later in *Cln3*^*-/-*^ mice than in *Ppt1*^*-/-*^ mice.

In general, the presented method can be used to perform non-invasive and longitudinal studies in mouse models of neurological diseases and might have important implications for diagnosis and monitoring of disease progression and therapeutic interventions.

## Material and methods

### Animals

Mice were kept at the animal facility of the Department of Neurology, University of Wuerzburg, under barrier conditions and at a constant cycle of 12 h in the light (< 300 lux) and 12 h in the dark. All animal experiments were approved by the Government of Lower Franconia, Germany.

*Ppt1*-deficient (*Ppt1*^*-/-*^) mice with disruption of exon 9 [13] and age-matched control (*Ppt1*^*+/+*^) littermates were on a uniform C57BL/6 genetic background. *Cln3*-deficient (*Cln3*^*-/-*^) mice with disruption of exons 2-6 and most of exon 1 [16] and age-matched control littermates (*Cln3*^*+/+*^) were on a Sv/129 genetic background. Genotypes were determined by conventional PCR using isolated DNA from tail biopsies following previously published protocols [13, 16]. Mice were screened for the confounding *Rd8* mutation in the *Crb1* gene as described [25]. Both mouse lines did not carry the described mutation.

We did not detect significant differences in the measured parameters between 18-month-old C57BL/6 and Sv/129 wildtype controls (not shown).

### Spectral domain Optical Coherence Tomography (OCT)

Mice were anesthetized by intraperitoneal injection of ketamine and xylazine, and their pupils were dilated by topical administration of 1 drop of 0.5% tropicamide eye drops (Mydrum; Bausch & Lomb) before image acquisition. Air-corneal interface refraction and corneal dehydration were prevented by applying artificial tears (Corneregel^®^ Fluid; Bausch & Lomb) and a custom-made polymethylmethacrylate hard contact lens (afocal, curvature: 1.7 mm, diameter: 3.2 mm; Cantor + Nissel). Mouse eyes were subjected to OCT imaging with a commercially available device (Spectralis OCT; Heidelberg Engineering) reaching a digital resolution of 3.9 μm and a measurable thickness change of 1 μm [26]. To adjust for the optical qualities of the mouse eye, we used a +25 diopter add-on lens (Heidelberg Engineering) that was placed directly in front of the camera unit. Imaging was performed with a proprietary software package (Heidelberg Eye Explorer, HEYEX, version 1.7.1; Heidelberg Engineering). The parameter for length of the reference pathway was manually adjusted using the “OCT debug window” to correct for the optical length of the scanning pathway with additional lenses. The combination of confocal scanning laser retinal fundus imaging and OCT allows for automatic real-time tracking (ART) of eye movements and real-time averaging of up to 100 images, profoundly reducing speckle noise. After the imaging procedure animals were kept warm, the contact lens was removed and cleaned and eyes were covered with Corneregel^®^ (Bausch & Lomb) until awakening of the mice. Resultant image files were used for analysis in the proprietary software or exported and processed using Photoshop CS3 (Adobe Systems). The analyzed retinal area was estimated based on previous observations [27], assuming that 30° field of view is subtended by 900 μm of retina.

For quantification of retinal thickness based on high-resolution OCT volume scans, we used the proprietary software. Briefly, each volume scan consisted of 49 high resolution B-scans with ART 20 recorded at 20° × 20° field-of-view centered on the optic disc. Volume scans were used to calculate interpolated retinal thickness maps. A segmented circular OCT grid was centered on the optic disc and retina thickness was calculated by averaging the values of the 4 outer areas.

Retinal composite layers were measured in high resolution OCT peripapillary circle scans with ART 100 centered on the optic disc. An investigator unaware of the genotype of the respective mice performed manual measurements at 600× and 1:1 μm view in the proprietary software. The following composite layers were well recognizable in all scans and therefore the retina was separated into 4 layers: 1) nerve fiber layer (NFL), ganglion cell layer (GCL) and inner plexiform layer (IPL); 2) inner nuclear layer (INL); 3) outer plexiform layer (OPL), outer nuclear layer (ONL) and inner/outer segments of photoreceptors (IOS); 4) retinal pigment epithelium (RPE) and choriocapillary complex (CC). Additionally, a combination of NFL, GCL, IPL and INL was defined as inner retina and a combination of OPL, ONL, IOS, RPE and CC was defined as outer retina. Five to ten measurements of each layer were performed along the width of the circle scan, avoiding measurements on top of major blood vessels.

### Histology, electron microscopy and immunofluorescence

Mice were euthanized and transcardially perfused with PBS containing heparin and subsequently with 2% paraformaldehyde (PFA) in PBS. Optic nerves and eyes were postfixed in the same solution for 1 h, dehydrated in 30% sucrose and frozen in OCT medium. 10 μm-thick cryo-sections of optic nerves and retinae were prepared and stored at -20°C until use.

Eyes were enucleated and postfixed in 4% PFA in PBS for 15 minutes and retinal flat mounts were prepared. Cresyl violet staining and quantification of Nissl-positive retinal ganglion cells was performed according to previously published protocols [18].

Immunohistochemical labeling of NeuN, Brn3a and non-phosphorylated neurofilaments in cryo-sections was performed as previously described [18].

For electron microscopy and correlation of OCT and histology, eyes with attached optic nerves were enucleated and postfixed in 4% PFA and 2% glutaraldehyde in cacodylate buffer over night. Tissue was osmicated, dehydrated and embedded in Spurr’s medium as described [18]. Semi-thin (0.5 μm) and ultra-thin (70 nm) sections through the optic nerve head were prepared and stained with methylene blue or lead citrate, respectively. Micrographs were acquired using an Axiophot 2 light microscope (Zeiss) with an attached CCD camera (Visitron Systems) or a Leo 906 E electron microscope (Zeiss) with a ProScan Slow Scan CCD camera. Measurements of the 4 defined retinal composite layers were taken on both sides of the optic nerve head mirroring the position of peripapillary OCT circle scans.

### Statistical analysis

Statistical analysis was performed using PASW Statistics 18 (SPSS, IBM) software. Shapiro-Wilk test was used to check for normal distribution of data. Parametric comparisons between thickness values of age-matched control mice and knockout mice were made by unpaired two-tailed Student’s *t*-test. Correlation of thickness measurements by OCT versus histology was assessed by Pearson’s correlation analysis.

## Results

### In vivo assessment of autofluorescent storage material and retinal thickness

**Figure 1:**
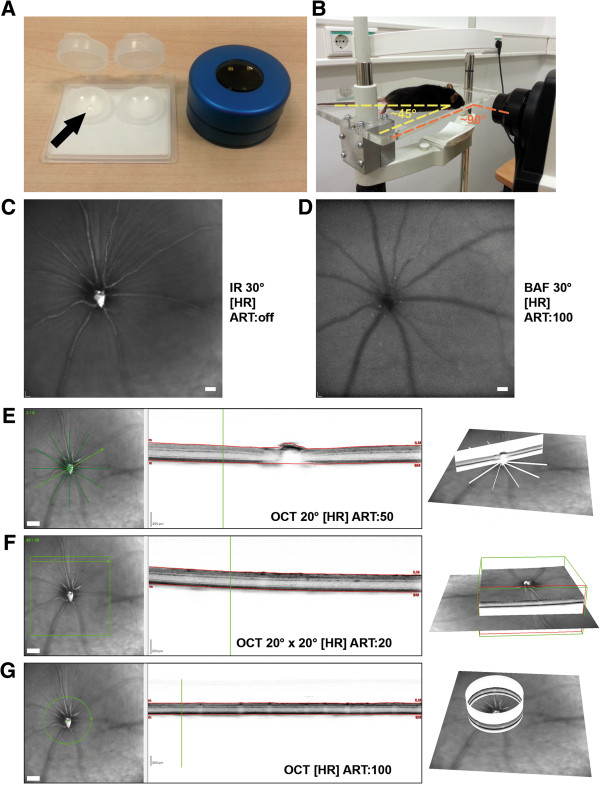
**Experimental setup and description of image acquisition settings. A**: Additional equipment for analysis of mouse eyes included a polymethylmethacrylate hard contact lens (arrow) and a +25 diopter add-on lens. **B**: Depiction of the custom-built rodent mount and positioning of the animal to allow imaging centered on the optic disc. **C**-**G**: Representative examples of the different imaging modes exemplarily shown for a *Ppt1* ^*+/+*^ mouse. **C**: infrared fundus image (IR), **D**: blue laser autofluorescence fundus image (BAF), **E**: OCT star scan (6 B-scans), **F**: OCT volume scan (49 B-scans), **G**: OCT peripapillary circle scan. Field of view, automatic real-time tracking (ART) and resolution settings are indicated in the corresponding images; HR, high resolution. For clarification, 3D-visualizations of OCT scans are depicted. Scale bars correspond to 50 μm of subtended retina in **C**, **D** and to 100 μm in fundus images in **E**, **F**, **G**.

We investigated mouse retinae using the commercially available Spectralis OCT device with slight modifications. A custom-made polymethylmethacrylate hard contact lens (Cantor + Nissel) and a +25 diopter add-on lens (Heidelberg Engineering) were used to adjust for optical qualities of the mouse eye (Figure 1A). A custom-made detachable and adjustable mount was built and anaesthetized mice were positioned at approximately 45° angle to the edge of the mount for imaging centered on the optic disc (Figure 1B). Examination was performed during approximately 10 to 15 minutes comprising the acquisition settings exemplarily shown for a *Ppt1*^*+/+*^ mouse (Figure 1C-G) at high resolution (HR).

**Figure 2:**
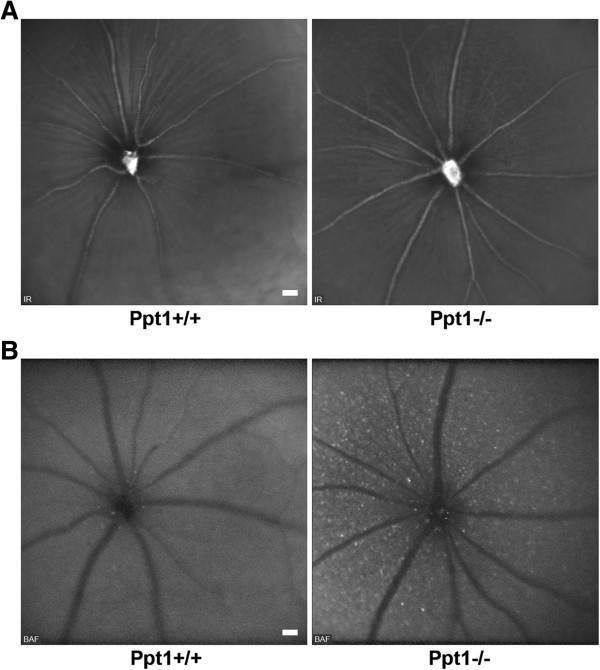
**Funduscopy of 7-month-old** ***Ppt1*** ^***+/+***^ **and** ***Ppt1*** ^***-/-***^ **mice. A**: IR fundus images revealed no obvious differences in general retinal appearance of *Ppt1* ^*-/-*^ mice in comparison with controls (*n* = 4 per group). Optic disc and major blood vessels were well recognizable. **B**: BAF funduscopy demonstrated accumulation of hyperfluorescent profiles in the inner retinal layers of *Ppt1* ^*-/-*^ mice in comparison with *Ppt1* ^*+/+*^ mice. Scale bars correspond to 50 μm of subtended retina.

In order to investigate retinal alterations in a mouse model of CLN1 disease, we analyzed 7-month-old *Ppt1*^*-/-*^ mice and age-matched *Ppt1*^*+/+*^ control littermates. At this age, our previous histological examination of retinal flat mounts demonstrated significant loss of retinal ganglion cells [18]. Infrared (IR) fundus images revealed no obvious alterations in general appearance of retinae from *Ppt1*^*-/-*^ mice compared with controls (Figure 2A). However, blue laser autofluorescence (BAF) fundus imaging revealed many spots of hyperfluorescence in *Ppt1*^*-/-*^ mice. Based on the focal plane and localization related to blood vessels, we concluded that these profiles accumulated in the inner retinal layers of *Ppt1*^*-/-*^ mice. Such hyperfluorescent profiles were rarely detected in *Ppt1*^*+/+*^ mice (Figure 2B).

**Figure 3:**
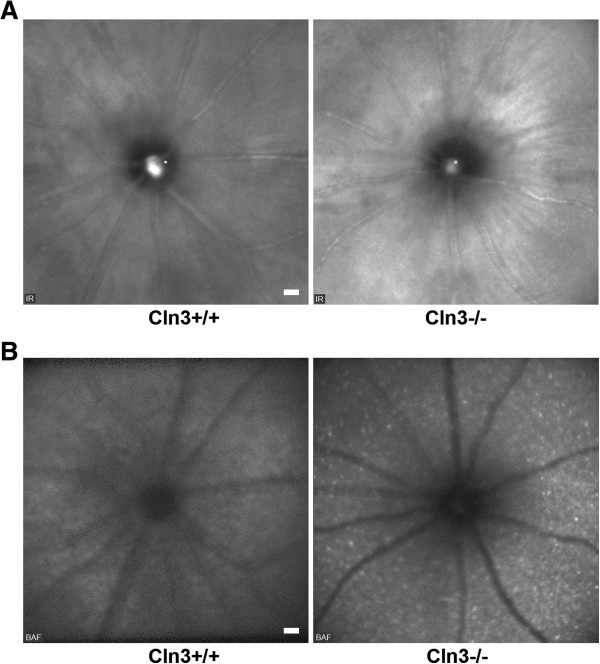
**Funduscopy of 16-month-old** ***Cln3*** ^***+/+***^ **and** ***Cln3*** ^***-/-***^ **mice. A**: IR fundus images revealed no obvious differences in general retinal appearance of *Cln3* ^*-/-*^ mice compared with controls (*n* = 4 per group). However, based on the different genetic background, both *Cln3* genotype groups showed brighter overall appearance of retinae in comparison with *Ppt1* genotype groups (see Figure 2A). Optic disc and major blood vessels were well recognizable. **B**: Accumulation of hyperfluorescent profiles in the inner retinal layers of *Cln3* ^*-/-*^ mice in comparison with *Cln3* ^*+/+*^ mice was obvious in BAF fundus images. Scale bars correspond to 50 μm of subtended retina.

Similarly, *Cln3*^*-/-*^ mice were investigated as a model of CLN3 disease. Since these mice were reported to show retinal pathology at advanced age [20, 22], we investigated 16-month-old *Cln3*^*-/-*^ mice and *Cln3*^*+/+*^ control littermates. Again, IR fundus images were not obviously different between the knockout and control mice (Figure 3A), while frequent hyperfluorescent spots were always observed in the inner retinal layers of *Cln3*^*-/-*^ but not *Cln3*^*+/+*^ mice (Figure 3B).

**Figure 4:**
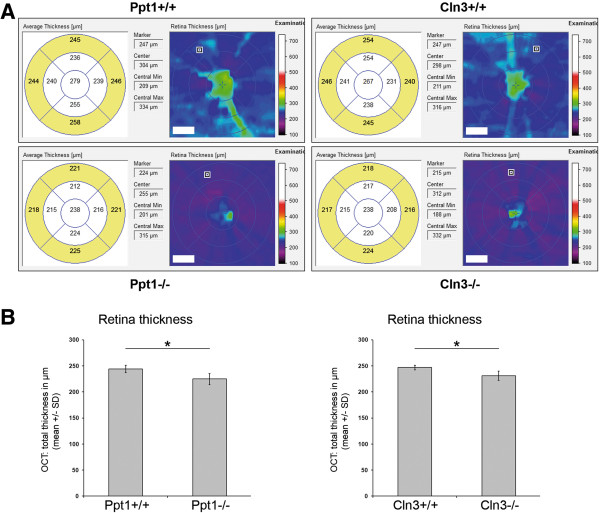
**Assessment of retina thickness in OCT volume scans. A**: Representative interpolated thickness maps from retinal volume scans of 7-month-old *Ppt1* ^*+/+*^ and *Ppt1* ^*-/-*^ mice and 16-month-old *Cln3* ^*+/+*^ and *Cln3* ^*-/-*^ mice. A segmented circular OCT grid was centered on the optic disc and retina thickness was calculated by averaging the values of the 4 outer areas (yellow filling). Scale bars correspond to 70 μm of subtended retina. **B**: Retina thickness was significantly reduced in *Ppt1* ^*-/-*^ and *Cln3* ^*-/-*^ mice compared to age-matched control littermates (*n* = 4 per group). Student’s *t*-test. * *P* < 0.05.

As a next step, the retinal thickness of 7-month-old *Ppt1*^*-/-*^ and 16-month-old *Cln3*^*-/-*^ mice and age-matched control littermates was determined using thickness maps generated from high-resolution volume scans (Figure 4A). The outer values from circular OCT grid subfields with the center located on the optic disc were averaged. In both *Ppt1*^*-/-*^ and *Cln3*^*-/-*^ mice, the mean retinal thickness was significantly reduced in comparison with age-matched control littermates (Figure 4B).

**Figure 5:**
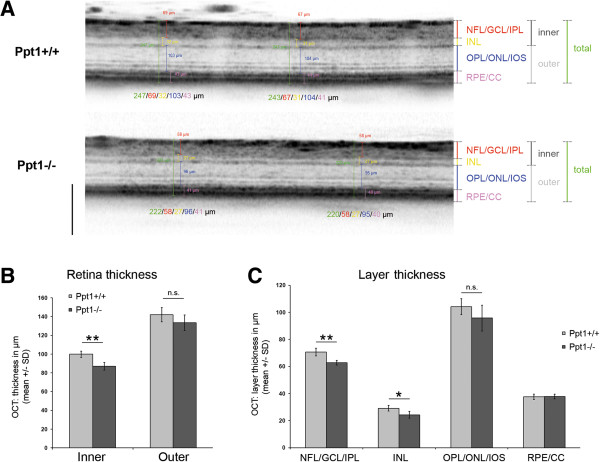
**Separation of peripapillary OCT circle scans from 7-month-old** ***Ppt1*** ^***+/+***^ **and** ***Ppt1*** ^***-/-***^ **mice into composite layers. A**: Representative excerpts of OCT circle scans from *Ppt1* ^*+/+*^ and *Ppt1* ^*-/-*^ mice with indication of the defined layers. Scale bar: 200 μm. **B**: Inner but not outer retina thickness was significantly reduced in *Ppt1* ^*-/-*^ mice (*n* = 4 per group). Student’s *t*-test. **P < 0.01. **C**: Significant thinning was detected in NFL/GCL/IPL and INL of *Ppt1* ^*-/-*^ mice, but not in other retinal layers. Student’s *t*-test. * *P* < 0.05, **P < 0.01. n.s. = not significant.

To further delineate thinning of retinal layers, manual measurements were performed on high resolution peripapillary circle scans by an investigator unaware of the genotype of the respective mice. The retina was subdivided into 4 composite layers as described in Material and Methods (Figure 5A). Inner and outer retina thickness were compared between 7-month-old *Ppt1*^*+/+*^ and *Ppt1*^*-/-*^ mice, revealing a significant thinning of the inner retina in *Ppt1*^*-/-*^ mice (Figure 5B). More precisely, there was significant thinning of both layer 1 (NFL/GCL/IPL) and 2 (INL), while layer 3 (OPL/ONL/IOS) showed a non-significant trend to thinning in *Ppt1*^*-/-*^ mice in comparison with controls (Figure 5C).

**Figure 6:**
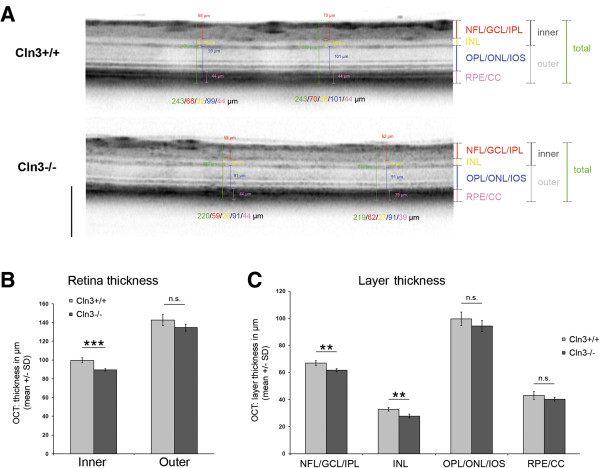
**Composite layer separation of peripapillary OCT circle scans from 16-month-old** ***Cln3*** ^***+/+***^ **and** ***Cln3*** ^***-/-***^ **mice. A**: Representative excerpts of OCT circle scans from *Cln3* ^*+/+*^ and *Cln3* ^*-/-*^ mice with indication of the defined layers. Scale bar: 200 μm. **B**: There was a significant reduction of the inner but not outer retina thickness in *Cln3* ^*-/-*^ mice (*n* = 4 per group). Student’s *t*-test. *** *P* < 0.001. **C**: Significant thinning was detected in NFL/GCL/IPL and INL of *Cln3* ^*-/-*^ mice, but not in other retinal layers. Student’s *t*-test. ** *P* < 0.01. n.s. = not significant.

Similar results were obtained with 16-month-old *Cln3*^*-/-*^ and age-matched *Cln3*^*+/+*^ mice. We detected a significant thinning of the inner retinal layers (layers 1 and 2) and a non-significant tendency for layer 3 (Figure 6A-C).

In summary, according to our investigations by BAF fundus imaging and OCT, accumulation of autofluorescent storage material and thinning of retinal layers as a marker of neuronal loss, are typical features of the inner retinal layers in *Ppt1*^*-/-*^ and *Cln3*^*-/-*^ mice.

### Comparison of OCT with histological analysis

**Figure 7:**
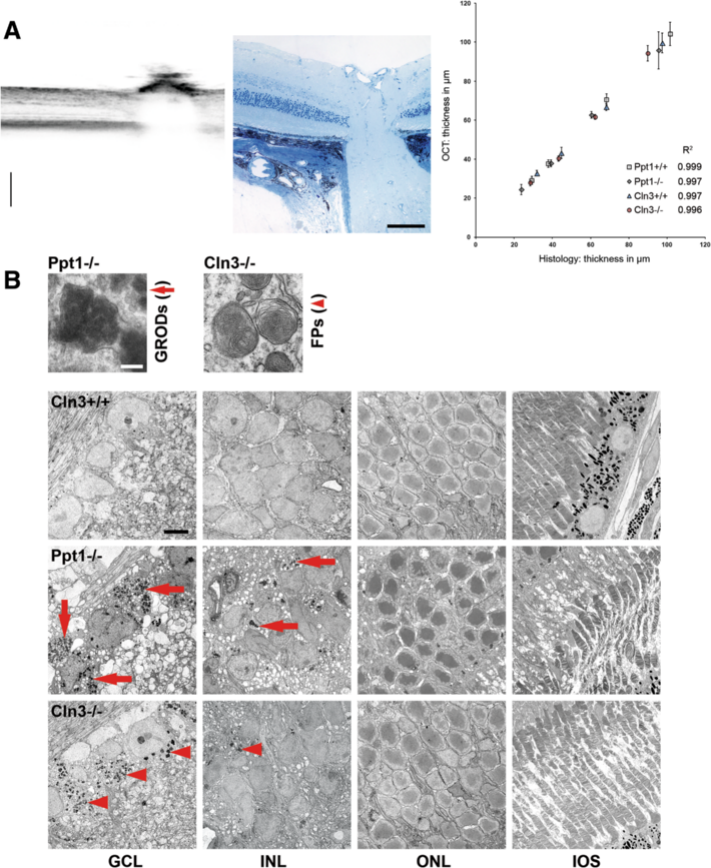
**Comparison of OCT with histological analysis. A**: Representative excerpt of an OCT B-scan through the optic nerve head and a corresponding micrograph of a 0.5 μm thick semi-thin section. Scale bars, 200 μm in OCT scan and 100 μm in micrograph. Thickness measurements of composite layers acquired by OCT correlated significantly with values measured in histological sections (*n* = 4 per group). Pearson’s correlation. *Cln3* ^*+/+*^, *P* < 0.01. *Cln3* ^*-/-*^, *P* < 0.01. *Ppt1* ^*+/+*^, *P* < 0.001. *Ppt1* ^*-/-*^, *P* < 0.01. Coefficients of determination (R^2^) are indicated for the different groups. **B**: Electron micrographs of retinal layers from 7-month-old *Ppt1* ^*-/-*^ and 16-month-old *Cln3* ^*+/+*^ and *Cln3* ^*-/-*^ mice. Accumulation of granular osmiophilic deposits (GRODs; arrows) was mostly observed in cells in the GCL and INL of *Ppt1* ^*-/-*^ mice, but not in *Ppt1* ^*+/+*^ (not shown) or *Cln3* ^*+/+*^ controls. Similarly, fingerprint profiles (FPs; arrowheads) were predominantly detected in cells in the GCL and INL of *Cln3* ^*-/-*^ mice, but not in *Ppt1* ^*+/+*^ or *Cln3* ^*+/+*^ mice. Scale bars: 0.2 μm and 5 μm.

To correlate the thickness values measured by OCT with values gained by histological examination, the investigated mice were dissected few days after initial OCT imaging. The corresponding retinae were processed for light and electron microscopy and the 4 defined composite layers were measured in retinal sections at similar positions as peripapillary circle scans by an investigator blinded to the genotype. Corroborating our findings, histological measurements and OCT measurements showed highly significant Pearson’s correlation with coefficients of determination (R^2^) close to 1 (Figure 7A).

The distribution of storage material in the retina was investigated by electron microscopy. Cytoplasmic inclusions in retinal cells presented as typical granular osmiophilic deposits in *Ppt1*^*-/-*^ mice and as fingerprint profiles in *Cln3*^*-/-*^ mice (Figure 7B), confirming previously described ultrastructural differences of the storage material [13, 16]. In both *Ppt1*^*-/-*^ and *Cln3*^*-/-*^ mice, the amount of storage material was clearly increased in comparison with age-matched control littermates showing little or no storage material (Figure 7B). The storage material was mostly observed in perikarya in the retinal ganglion cell layer and, at lower amounts, in perikarya in the inner nuclear layer of both knockout models. Cells in the outer nuclear layer or inner and outer segments of photoreceptors showed no obviously increased accumulation of storage material (Figure 7B). Thus, both techniques, OCT and EM reveal a spatially corresponding deposition of storage material, confirming the reliability of the non-invasive autofluorescence imaging approach.

### Axonal perturbation and loss of retinal ganglion cells in Cln3^-/-^ mice

OCT imaging indicated significant degenerative alterations in the inner retina of *Cln3*^*-/-*^ mice at advanced age. This finding is in accordance with reports demonstrating optic nerve pathology in *Cln3*^*-/-*^ mice [22] and reminiscent of *Ppt1*^*-/-*^ mice in which we have reported significant loss of retinal ganglion cells [18]. To confirm the observed thinning as a sign of neurodegeneration also in this model, we investigated 18-month-old *Cln3*^*-/-*^ mice and age-matched *Cln3*^*+/+*^ littermates regarding integrity of retinal ganglion cells.

**Figure 8:**
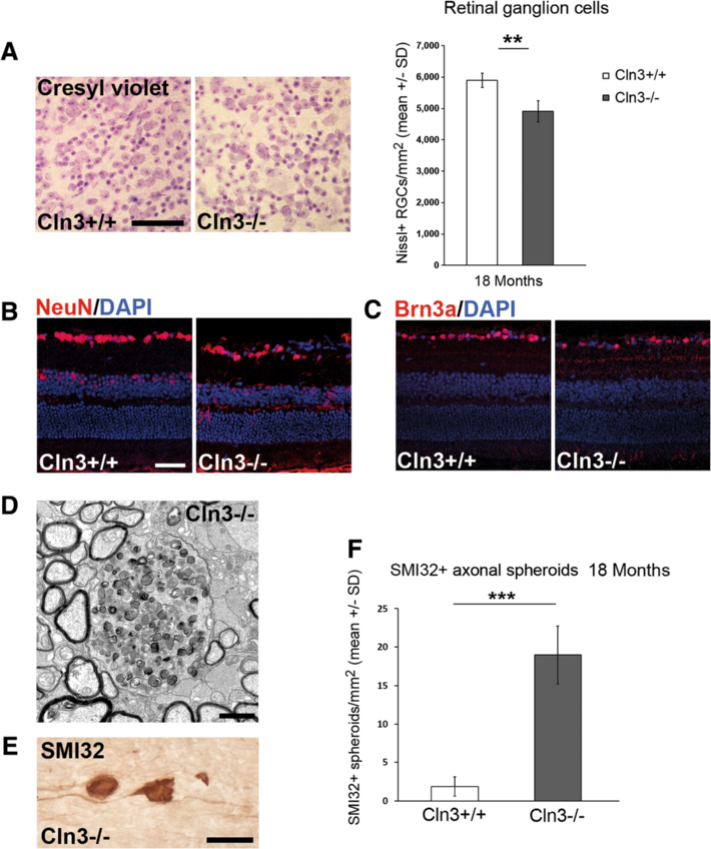
**Loss of retinal ganglion cells and axonal perturbation in optic nerves of** ***Cln3*** ^***-/-***^ **mice. A**: Cresyl violet staining of retinal flat mount preparations and quantification of Nissl + retinal ganglion cells in 18-month-old *Cln3* ^*+/+*^ and *Cln3* ^*-/-*^ mice (*n* = 4 per group). Scale bar: 50 μm. **B**: NeuN and **C**: Brn3a immunohistochemistry on retinal sections from 18-month-old *Cln3* ^*+/+*^ and *Cln3* ^*-/-*^ mice. Scale bar, 30 μm. A loss of NeuN + or Brn3a + retinal ganglion cells in *Cln3-/-* mice was obvious. **D**: Representative electron micrograph of optic nerve cross-sections from 18-month-old *Cln3-/-* mice. Axonal spheroids with accumulation of organelles and dense bodies were frequently observed. **E**: Immunohistochemistry using antibodies against non-phosphorylated neurofilaments (SMI32; brownish precipitate) in longitudinal optic nerve sections of 18-month-old *Cln3* ^*-/-*^ mice visualizes axonal spheroids. Scale bar: 30 μm. **F**: Quantification revealed that SMI32+ axonal spheroids are barely detectable in optic nerves of *Cln3* ^*+/+*^ mice but accumulate in 18-month-old *Cln3* ^*-/-*^ mice (*n* = 4 per group). Student’s *t*-test. ***P < 0.001.

Retinal flat mounts were prepared, stained with cresyl violet, and Nissl-positive neurons were quantified in the retinal ganglion cell layer. We detected a significant loss of retinal ganglion cells in *Cln3*^*-/-*^ mice in comparison with age-matched *Cln3*^*+/+*^ control littermates (Figure 8A). Loss of retinal ganglion cells was also confirmed by immunohistochemical detection of the neuronal marker NeuN and the transcription factor Brn3a in cryo-sections. For both markers, *Cln3*^*-/-*^ mice presented with fewer immunoreactive cells in the ganglion cell layer in comparison with *Cln3*^*+/+*^ mice (Figure 8B,C).

Our previous investigation of *Ppt1*^*-/-*^ mice revealed that impaired axonal integrity and axonal degeneration precede neuronal loss and are accompanied by axonal swelling and spheroid formation [18]. We therefore analyzed optic nerves of *Cln3*^*-/-*^ mice at 18 months of age by electron microscopy and immunohistochemistry against non-phosphorylated neurofilaments (Figure 8D,E) and detected axonal spheroids that appeared morphologically similar to those in younger *Ppt1*^*-/-*^ mice. Immunohistochemical quantification of these spheroids confirmed a significantly increased amount of SMI32-positive degenerating axons in optic nerves of *Cln3*^*-/-*^ mice in comparison with *Cln3*^*+/+*^ mice, in which such spheroids were rarely detected (Figure 8F).

## Discussion

To our knowledge, this is the first study using OCT as a method for non-invasive assessment of retinal degeneration in mouse models of infantile and juvenile neuronal ceroid lipofuscinosis. Blue laser autofluorescence fundus imaging faithfully revealed the typical accumulation of autofluorescent storage material in the inner retinal layers of both knockout mouse models. Moreover, OCT scans revealed a thinning of the retina in both models, confirming the histologically observed retinal degeneration at distinct age of onset, as reported previously [18–20, 22]. Delineation of different retinal composite layers by non-biased manual measurements demonstrated that this thinning is predominantly due to a reduced thickness of the inner retinal layers, correlating well with subsequent histological evaluation and investigation of retinal ganglion cell survival and axonal integrity.

These findings display some common features in retinae of *Ppt1*^*-/-*^ and *Cln3*^*-/-*^ mice. A predominant dysfunction of postsynaptic (inner) retinal neurons has also been indicated by electroretinographic analysis of *Ppt1* and *Cln3* mutant mice, as mostly b-wave amplitudes were affected [17, 21, 23]. However, a trend towards thinning was also seen in the outer retina, not reaching statistical significance. Based on our histological examinations, this lack of statistical significance is most likely not due to a limited resolution of the OCT technique, but might rather be due to the limited sample sizes and/or the age of the mice investigated. Of note, in mouse models of other CLN disease variants, as for instance in *Cln6* mutant mice, a predominant degeneration of the outer retina has been demonstrated [28]. Similarly, there is significant variety in the presentation of retinal defects also in patients suffering from distinct CLN forms [29]. It remains to be determined why there seems to be a predominant degeneration of distinct retinal layers in models of different CLN disease types. Some disparities regarding the progression of neuronal loss in specific brain regions have also been described in models of different CLN disease forms in other studies [30].

The feasibility of OCT for non-invasive imaging of storage material and neurodegeneration has important implications for longitudinal imaging in animal studies. Our previous observations have shown that loss of retinal ganglion cells is a reliable indicator of neuron loss in other brain regions and that the retina can be used as a surrogate tissue to monitor disease severity at least in a model of CLN1 disease [18]. This also seems to be valid for *Cln3*^*-/-*^ mice, as retinal alterations (this study and unpublished observations) and regional brain atrophy [31] occur only at advanced age. Moreover, visualization of autofluorescent storage material might provide a good read-out to validate strategies aimed at correcting the primary disease cause, such as enzyme replacement therapy. Thus, OCT seems to be an ideal tool to measure disease progression and to assess efficacy of putative therapeutic approaches in rodent models. According to presently unpublished and preliminary observations, OCT analysis is of sufficient sensitivity to confirm the previously described ameliorating effect of *Rag1* deficiency regarding retinal ganglion cell loss in *Ppt1*^*-/-*^ mice [18], demonstrating the feasibility of this approach to assess disease-modifying strategies. The consequent option of the present technique to examine the same animal at different time-points allows for exact determination of kinetics of disease progression and other disease-specific parameters. In addition to the presented substantial advantages of the technique, non-invasive imaging reduces biological variation (refinement) and eventually numbers of experimental animals (reduction), which has important ethical implications regarding research in animal models.

Moreover, the presented method might be of high relevance for studies or trials in patients. Non-invasive imaging techniques revealing typical alterations associated with CLN diseases (storage material, neurodegeneration) might not only guide in diagnostics, but are also important to determine disease status and severity and control efficacy of therapeutic interventions. As the perspectives for such treatment approaches become increasingly more promising the demand for efficient non-invasive techniques to judge their efficacy is constantly growing [32, 33].

Finally, non-invasive assessment of specific retinal layers appears to emerge as a powerful tool to monitor disease severity also in other more common neurological disorders and their corresponding models. For example, in line with our findings, thinning of inner retinal layers is also associated with a loss of retinal ganglion cells in a mouse model of multiple sclerosis [34] and is a typical feature in multiple sclerosis and Alzheimer’s patients [3, 35, 36]. There is already a wide spectrum of diseases and corresponding animal models that can be monitored using OCT, and future studies will most likely extend this spectrum to include more neurodegenerative disorders of primarily non-ophthalmologic origin.

## Conclusions

We demonstrate the feasibility of OCT to assess distinct alterations related to disease severity in the retina of two mouse models of neuronal ceroid lipofuscinosis *in vivo*. Reflecting accompanying conventional histological investigations, both accumulation of autofluorescent storage material and retinal thinning indicative of neurodegeneration mainly occur in the inner retina of the analyzed mice. The non-invasive method allows for longitudinal studies in experimental models, reducing the number of animals used for research. Moreover, it might have important implications for diagnostic evaluation of disease progression and therapeutic efficacy in patients. The presented method might emerge as an important tool to monitor neuronal alterations not only in CLN diseases, but in many neurological disorders characterized by widespread axonal perturbation in the CNS.

## Abbreviations

CLN: Ceroid lipofuscinosis neuronal
PPT1: Palmitoyl protein thioesterase 1
OCT: Optical coherence tomography
BAF: Blue laser autofluorescence
IR: Infrared
ART: Automatic real-time tracking
NFL: Nerve fiber layer
GCL: Ganglion cell layer
IPL: Inner plexiform layer
INL: Inner nuclear layer
OPL: Outer plexiform layer
ONL: Outer nuclear layer
IOS: Inner/outer segments of photoreceptors
RPE: Retinal pigment epithelium
CC: Choriocapillary complex.
